# Relevant Information and Informed Consent in Research: In Defense of the Subjective Standard of Disclosure

**DOI:** 10.1007/s11948-016-9755-4

**Published:** 2016-01-20

**Authors:** Vilius Dranseika, Jan Piasecki, Marcin Waligora

**Affiliations:** 1 REMEDY, Research Ethics in Medicine Study Group, Department of Philosophy and Bioethics, Faculty of Health Sciences, Jagiellonian University Medical College, Michalowskiego 12, 31-126 Kraków, Poland; 2 Department of Logic and History of Philosophy, Faculty of Philosophy, Vilnius University, Vilnius, Lithuania

**Keywords:** Informed consent, Relevant information, Research ethics, Manipulation, Disclosure

## Abstract

In this article, we seek to contribute to the debate on the requirement of disclosure in the context of informed consent for research. We defend the subjective standard of disclosure and describe ways to implement this standard in research practice. We claim that the researcher should make an effort to find out what kinds of information are likely to be relevant for those consenting to research. This invites researchers to take empirical survey information seriously, attempt to understand the cultural context, talk to patients to be better able to understand what can be potentially different concerns and interests prevalent in the target population. The subjective standard of disclosure should be seen as a moral ideal that perhaps can never be perfectly implemented but still can and should be used as a normative ideal guiding research practice. In the light of these discussions, we call for more empirical research on what considerations are likely to be perceived as relevant by potential research participants recruited from different socio-economic and cultural groups.

## Disclosure of Relevant Information as a Prerequisite to Valid Consent

One of the most firmly established requirements of research ethics is that a potential research participant cannot validly consent to take part in biomedical research unless she/he was adequately informed about the study (World Medical Association 2013; Council for International Organizations of Medical Sciences 2002; U.S. Food and Drug Administration 2015; United Nations Educational, Scientific and Cultural Organization 2005; European Parliament and of the Council of the European Union 2014). Still, there are controversies concerning the explication and application of this requirement. Many commentators often stress that it is not realistic to communicate all study details to potential participants (O’Neill 2002: 156; Beauchamp 2010: 65; Veatch 1978: 26; Ingelfinger 1972: 465). Primarily, because much of the information about the proposed study would likely be too technical or too detailed for most of the participants to comprehend. In addition to that, a lot of the information is likely to be of no interest whatsoever to the participants themselves.

On the other hand, it also seems not entirely satisfactory to provide in advance a complete closed list of items that must be described and communicated to potential research subjects in any kind of research study. Assuming the full list of necessary consent elements is provided (as described, for example, in Council for International Organizations of Medical Sciences 2002: Guideline 5; U.S. Food and Drug Administration 2015: 21CFR50.25; or European Parliament and of the Council of the European Union 2014: Article 29), the question of the level of detail remains. Furthermore, there is always a chance that something important to participants will be missed, since research as an enterprise is essentially open-ended. Lists, if they cover many items, also may result in long and burdensome consent forms and procedures.

A familiar solution is to resort to some notion of *relevance*, *importance*, or *materiality* of information. Let us provide some examples:

“The requirement that consent be informed requires that no *relevant* information be deliberately withheld from the one who consents” (Brock 1980; emphasis ours).

“If the information covers all issues that are *relevant* for a person’s choice, then that person’s consent is appropriately informed” (Hansson et al. 2006: 266; emphasis ours).

“The subjects need not be told everything, but they do have to be told the *important* things” (Katzner 1979: 46).

“Informed consent can never strictly speaking be *fully* informed: the point is rather that it should be *relevantly* informed” (O’Neill 2002: 156).

This solution can also take the shape of an open list of the most important items. This list is then finished by adding that any other relevant or important aspects of the study must be described as well. For example:

“[E]ach potential subject must be adequately informed of the aims, methods, sources of funding, any possible conflicts of interest, institutional affiliations of the researcher, the anticipated benefits and potential risks of the study and the discomfort it may entail, post-study provisions and any *other relevant* aspects of the study.” (World Medical Association 2013: Art 26; emphasis our).

This resort to the notion of relevance seems to be a step in the right direction. It allows the researchers (and research ethics committees) to focus only on *some* rather than *all* information and, in addition, promises to retain all non-trivial, non-redundant, important information. This helps to promote the decisional autonomy of research participants.

Resorting to the notion of relevance turns the requirement of disclosure into a claim that it is not permissible to withhold from potential research participants information that is *relevant to them*. And here it is tempting to understand relevant information as information that is actually perceived by the potential research participant herself/himself to be important to her/his choice whether to participate in research. However, it can be argued that relevance should be understood in a broader sense which does not require the participants themselves to be subjectively aware of what would constitute relevant considerations to them. Thus relevance is not limited to what is actually perceived as relevant but also extends to what would counterfactually be perceived as relevant, i.e. information that, *if* made salient to the potential research participant, *would be* perceived by her to be important to her choice as to whether to participate in the research. This understanding of relevance is commonplace in the literature, even if sometimes wording makes it difficult to disambiguate between the two readings presented above (Helgesson 2010: 31; 2012: 45; Feinberg 1984: 307; Faden and Beauchamp 1986: 303).

Often these two ways of understanding relevance will issue the same verdict but sometimes they may differ. For example, when some piece of information would, if presented, influence the choice of the participant but the participant is not presently aware of that—perhaps these considerations simply never crossed her/his mind. It still may be the case that if this piece of information were brought to her/his attention, she/he would find it relevant.

This idea is key to defense of informed consent in terms of autonomy and self-determination. The argument goes as follows: once the researcher comes to believe that some piece of information is—either actually or counterfactually—relevant to the participant, the researcher should disclose this information. Failing to do so will result in disrespect for the participant’s autonomy and may also result in deceit and, perhaps, manipulation. As stated by Bromwich and Millum, “When the researcher withholds information about a risk that she reasonably believes would be relevant to the prospective participant’s enrolment decision, she arrogates his role as agent by determining what information he gets to consider” (2013; see also Feinberg 1984: 307).

In what follows we discuss the notion of relevance in the light of different standards of disclosure of information to the potential study participants and also discuss ways in which the researchers can determine what kinds of information are likely to be relevant for those consenting to research. This leads us to call for more empirical research on what considerations are likely to be perceived as relevant by potential research participants recruited from different socio-economic and cultural groups.

## On Knowing What’s Relevant

Even if a broad understanding of relevance, as described in the previous sections, is agreed upon, this leaves us with the question of the standards of its implementation. Traditionally, there are three competing standards: *professional standard*, *reasonable person standard*, and *subjective standard* (Faden and Beauchamp 1986; Beauchamp 2010; Morin 1998; the latter standard is sometimes called *individual standard*). Mixed standards involving elements of these three standards can also be found in the literature. Professional standard refers to informational obligations as understood by the professional community and as required by internationally-agreed rules. Reasonable person standard, on the other hand, invites us to ponder on what considerations would be relevant for a reasonable person. The biggest moral problem with the first two standards is that they ignore the possibility that competent people may have different values, different experiences and concerns than the idealized type-person of professional standard or reasonable person standard. This problem becomes especially vivid in cross-cultural research or research on social and demographic groups that may differ significantly from the professionals applying the standard.

The subjective standard—the standard that requires one to attempt to understand what information is relevant to particular individuals, however, aims to honor these potential differences in normatively significant preferences. Thus Thomas May claims that the subjective standard “reflects, at the fundamental level, the liberal ideal of protecting an individual’s right to author his or her own life” (2002: 19). Considerations like these also led some commentators to declare that the subjective standard is the preferred *moral* standard (Beauchamp and Childress 2001: 83).

One may object that individual participants may have irrational beliefs. This, however, invites the question of the ability to provide consent. If the nature of what seem to be irrational beliefs is such that they render the potential participant unable to provide consent, this has to be formally assessed. If, however, the participant is able to provide consent, then even irrational (from the point of view of the researcher) concerns should be addressed. This is especially important in cross-cultural encounters. Examples of unacceptable (to some) practices may include treating donated tissues or organs as items for display, commercialization of samples, research on human embryos; research on non-medical, say cosmetic, products, research on contraception or mental illness, forensic access to samples and information stored for research purposes (O’Neill 2002: 160; Dranseika et al. 2015).

The major problem with the subjective standard is often thought to be epistemic—the researcher is not in a position to know what considerations are relevant to a given individual or a group. There is always a possibility that the researcher, even after an honest effort, fails to recognize that some pieces of information are relevant—either actually or counterfactually—to the potential participant. Another way to put this is that the morally significant criterion of relevance is externalistic—whether some piece of information is relevant depends on circumstances that sometimes may be inaccessible from the researcher’s perspective. If this standard is accepted, however, the researcher should make efforts to recognize relevant information—it is the researcher who decides based on her reasonable beliefs about what is considered relevant by participants [This can be described as metarepresentation: the researcher is expected to act on her beliefs about the beliefs of the participants. Metarepresenational capacities can often be helped by empathic ability. A researcher can be expected to make an effort to imagine what her subject(s) might consider important. The following point made by Benjamin Freedman illustrates both points: “The proper test of whether a given piece of information needs to be given is, then, whether the physician, knowing what he does about the patient/subject, feels that that patient/subject would want to know this before making up his mind” (1975: 34).]. The same expectation extends to members of research ethics committees—they can also be expected to make honest efforts to assess whether informed consent procedures described in the research protocol in question include all the information that is likely to be relevant to potential research participants.

How can the researcher come to know what are the relevant considerations? Other standards than the subjective standard may serve as a useful starting point where the subjective standard is not particularly helpful. The professional and the reasonable person standards as well as available formal regulatory standards may serve as proxies that allow us to tap into individual normative preferences. For example, information can be based on a reasonable person standard—even if this standard here is better applied as setting necessary, not sufficient, conditions of relevance. This involves some armchair considerations, perhaps mostly based on what the researcher herself finds to be relevant. This is good as a beginning point, but other sources can add additional pieces of relevant information. Professional standard and reasonable person standard derive their normative force from their potential to mimic the subjective standard—they may serve as a useful heuristic when the application of the subjective standard faces difficulties. Still, the subjective standard should have the normative priority—once it is revealed that something is declared relevant from the point of view of the first two standards but irrelevant from the third one, it may well be justifiable to omit this information. This would perhaps even make the disclosure procedure more focused. More importantly, however, if something is found to be relevant based on the subjective standard while the same thing is found to be irrelevant by applying either of the other two standards, the subjective standard should prevail since it reveals what is relevant to the particular individual in question. As indicated by Veatch:

“If the goal is providing enough information for adequate self-determination, surely the reasonable person standard is not adequate for such subjects. If there is any reason to believe that the particular patient or subject wants more information than the reasonable citizen, then the patient or subject’s own standard of certainty must apply” (1978: 32).

Let us mention several potential sources from which researchers can learn what is likely to be relevant to particular research participants.

First, researchers sometimes just happen to know some facts about the individual in question. Maybe they have information from earlier chats with potential participants on their ethical or religious convictions; perhaps they are aware of their phobias etc. This is not a systematic source of information and often no such information will be available. However, *if* the researcher happens to learn about some relevant facts, they should be honored. (Such evidence is more likely to be available in research in clinical settings, where prolonged familiarity with potential research participants is more likely to occur.)

Second, sometimes simply asking the potential participant or giving the participant the opportunity to ask questions may reveal some facts about relevance. This seems to be one of the reasons why potential participants are given the chance and encouraged to ask questions. This invites seeing participants as research partners rather than passive recipients of research procedures (Katz 1973; Levine 1988).

Many of the previous debates on the application of the subjective standard were based on discussions of such highly individualized information. What we want to add to the debate is that resorting to empirical studies is a powerful and not sufficiently appreciated way to make the application of the subjective standard more tractable.

## Empirical Research on Relevant Considerations

There are systematic empirical ways to learn about potentially relevant considerations. However, as concluded in a recent systematic review of literature (covering 14 studies published between 1950 and 2010), “There is little empirical evidence of what information potential participants want to know about research when they are making the decision to take part … The limited empirical evidence available suggests that potential participants may have very different information needs” (Kirkby et al. 2012: 1; newer studies not included in this review include Gillies et al. 2013; Kirkby et al. 2013; Coors et al. 2015). When little evidence is available, however, it is often coarse-grained and does not cover finer details of information needs.

Even though there is relatively little research directly designed to recover what types of information are perceived to be relevant by research participants and more such research is certainly called for, sometimes it is possible to obtain indirect evidence about potentially relevant considerations from other types of research. These include studies of public opinion on various aspects of research and research participation such as privacy and confidentiality, preferences toward consent types, trust in researchers and healthcare system, policies for return of findings and dissemination of results.

Certain concerns may be more prevalent in some societal groups than others and thus research on the effects of such demographic factors as age, sex or ethnicity/race can be very helpful in assessing information needs of research participants recruited from such groups. The same is true of assessing the information needs of specific patient groups in clinical research. As noted by Klaus Hoeyer, “there is no reason to presume universal agreement among donors as a homogenous group, or to think that universal standards will serve local interests and concerns. It would be more productive to pay attention to potential differences among donors” (2010: 346). For example, in some societies certain ethnic groups may be more concerned than others about access to their biological samples by law-enforcing institutions (Kaufman et al. 2009; other studies looking at the influence of demographic and cultural variables include Platt et al. 2014; Hull et al. 2008; Fong et al. 2004; Goldenberg et al. 2011; Wendler and Emanuel 2002; Ma et al. 2012; see also Hoeyer 2010; Hussain-Gambles et al. 2004).

We believe that there is a need to conduct more empirical research on what considerations are potentially relevant to research participants. If some aspects are found to be relevant to a sizeable proportion of the public or some more circumscribed group, then the researcher, planning to recruit her/his research participants from the population in question, can be expected to include these aspects into the content of disclosure requirements. It is important to note here that placing strict numerical values as to what proportion of the public should count as ‘sizeable’ may be difficult to justify.

A related but not identical source of hints about potentially relevant considerations is cultural knowledge, which is especially important in conducting research outside familiar cultural contexts. This kind of knowledge concerning potentially relevant information can come from sociological research but it also can result from anthropological research or from experience of different research or healthcare organizations that have experience in the cultural contexts in question. Requirements of cultural sensitivity that are present in a variety of international documents are of precisely this variety. For example, the Council for International Organizations of Medical Sciences states that:

“The ability to judge the ethical acceptability of various aspects of a research proposal requires a thorough understanding of a community’s customs and traditions. The ethical review committee in the host country, therefore, must have as either members or consultants persons with such understanding; it will then be in a favourable position to determine the acceptability of the proposed means of obtaining informed consent and otherwise respecting the rights of prospective subjects as well as of the means proposed to protect the welfare of the research subjects.” (2002: 31).

Information on group identity supplemented with empirical information on concerns more typical of this group or another may serve as an extremely useful means to apply the subjective standard (see also Minnow 1997). If research participants are sampled from a given group and there is some information available about what is particularly relevant to this group, the subjective standard requires the use this information. What constitutes a relevant consideration for a given group may be non-obvious for a researcher. For example, Megan Fong and her colleagues discuss the following concerns in their study showing that Native Hawaiians are relatively more cautious about donating biological samples for research:

“Additionally, some Native Hawaiians maintain traditional beliefs that require certain ritual practices surrounding tissue and body parts, especially of the deceased, and specifically forbid the desecration of placentas, bones, hair, fingernails, and excrement. While western minds may view these biological specimens as inconsequential and disposable, Native Hawaiians believe that they contain *mana* or the very life force of the individual” (2004: 158; for more on potential differences between perspectives of researchers and participants on meaning and significance of blood and other biological materials see (Drabiak-Syed 2010)).

Moral prevalence of the subjective standard obliges the researchers to make some effort to investigate and find out what is it that has to be disclosed. This does not mean that the researchers are obliged to do empirical research on what may be relevant to their target populations or even engage in extensive literature searches on these topics. Still, this places an obligation on them to be open to hints and suggestions once they appear—either in academic literature on what types of considerations can be relevant to different groups or perhaps in sharing experience about consent procedures with their colleagues, involved in similar research on similar populations.

Often, researchers will not be aware that some information is in fact relevant to their potential participants. However, the subjective standard does not presuppose omniscience on the part of the researcher. It puts a moral expectation on researchers that they try to learn more about the concerns of their study population as well as particular research subjects. It also asks not to ignore relevant information once they learn that it is relevant. This is a handy approach that helps the recognition of at least some of the information that should not be omitted, even if it is not a reliable guide to recognize all such information. Furthermore, it is not only researchers who should be interested in what is likely to be perceived as relevant by research participants. Members of research ethics committees should also be expected to address the issue of relevant considerations. Involving representatives of local communities may help to identify potentially relevant information and thereby contribute to the quality of informed consent forms and processes. Finally, endorsement of the subjective standard motivates empirical research on what considerations are potentially relevant in different cultural contexts.

The relevant considerations approach is essentially contextual; it is not a “one-size-fits-all approach” (Helgesson 2012: 44). Different types of information will be relevant to different potential participants. And researchers are expected to be sensitive to these contextual cues and to be willing to question their own implicit assumptions about what constitutes research harms and risks. This is especially vivid in dealing with vulnerable populations and/or people from a different cultural background (Drabiak-Syed 2010). According to this approach, researchers are under informational obligation to disclose all the information they have reasons to believe can be relevant to potential research participants in a given context.

## Concluding Remarks

All information that is relevant to potential research participants should be disclosed to them. This, however, does not presuppose that researchers will always be in a position to recognize what information is relevant in a given situation. In this article we claim that researchers should make an effort to learn what considerations are potentially relevant to their target population or individual. This invites researchers to take empirical survey information seriously, to attempt to understand the cultural context, talk to patients, make an honest effort to understand what can be potentially different concerns and interests prevalent in the target population. And once the researcher knows or has good reasons to believe that given information is likely to be relevant to the potential participants, he/she has an informational obligation to disclose it in good faith. The subjective standard of disclosure should be seen as an ideal that perhaps can never be perfectly implemented but still can and should be used as a normative ideal guiding research practice. In the light of these discussions, we call for more empirical research on what considerations are likely to be perceived as relevant by potential research participants recruited from different socio-economic and cultural groups.
